# DrawnNet: Offline Hand-Drawn Diagram Recognition Based on Keypoint Prediction of Aggregating Geometric Characteristics

**DOI:** 10.3390/e24030425

**Published:** 2022-03-19

**Authors:** Jiaqi Fang, Zhen Feng, Bo Cai

**Affiliations:** Key Laboratory of Aerospace Information Security and Trusted Computing, Ministry of Education, School of Cyber Science and Engineering, Wuhan University, Wuhan 430072, China; fangjiaqi@whu.edu.cn (J.F.); fengzhen@whu.edu.cn (Z.F.)

**Keywords:** diagram recognition, offline recognition, object detection

## Abstract

Offline hand-drawn diagram recognition is concerned with digitizing diagrams sketched on paper or whiteboard to enable further editing. Some existing models can identify the individual objects like arrows and symbols, but they become involved in the dilemma of being unable to understand a diagram’s structure. Such a shortage may be inconvenient to digitalization or reconstruction of a diagram from its hand-drawn version. Other methods can accomplish this goal, but they live on stroke temporary information and time-consuming post-processing, which somehow hinders the practicability of these methods. Recently, Convolutional Neural Networks (CNN) have been proved that they perform the state-of-the-art across many visual tasks. In this paper, we propose DrawnNet, a unified CNN-based keypoint-based detector, for recognizing individual symbols and understanding the structure of offline hand-drawn diagrams. DrawnNet is designed upon CornerNet with extensions of two novel keypoint pooling modules which serve to extract and aggregate geometric characteristics existing in polygonal contours such as rectangle, square, and diamond within hand-drawn diagrams, and an arrow orientation prediction branch which aims to predict which direction an arrow points to through predicting arrow keypoints. We conducted wide experiments on public diagram benchmarks to evaluate our proposed method. Results show that DrawnNet achieves 2.4%, 2.3%, and 1.7% recognition rate improvements compared with the state-of-the-art methods across benchmarks of FC-A, FC-B, and FA, respectively, outperforming existing diagram recognition systems on each metric. Ablation study reveals that our proposed method can effectively enable hand-drawn diagram recognition.

## 1. Introduction

Hand-drawing is considered as one of the most natural and efficient ways for humans to record information. As early as in ancient Egypt, in order to convey information, Egyptians invented some handwritten symbols and carved them on stone walls with tools to record. These handwritten symbols are the predecessors of today’s characters [1]. Nowadays, due to the widespread usage of smartphones and electrical whiteboards, recording information in digital devices has become a popular choice for its convenience. As a result, handwritten text recognition such as words and mathematical formulas has been intensively studied over the last few decades and widely applied in many fields  [2,3,4,5]. However, the recognition and analysis targeted at hand-drawn diagrams, such as flowcharts, circuits, and music scores, are still challenging because of their complex two-dimensional structures and symbol contour variations [6,7].

A hand-drawn diagram is undoubtedly a powerful expressing way that can assist with the illustration of people’s ideas. They contain self-explanatory symbols and can expand their styles freely. Meanwhile, research for diagram recognition has a long history. In this area, one of the most wide-studied tasks is the recognition of hand-drawn flowcharts, which makes the illustrations of programs or structural objects very intuitive. Flowchart recognition can be divided into two basic cliques: online and offline recognition. Online recognition refers to the input being a sequence of strokes captured by an ink input device such as a tablet [2,5,8,9,10]. Each stroke is defined as a press-down and lift-up writing act using pen, which is tracked as a series of point coordinates [2,5,8,9,10]. Off-line recognition emphasizes that input turns out a digital image containing symbols of strokes presented as pixels [2,6,7,11].

Because of popularization and convenience of modern ink input devices, a lot of attention has been given to the online handwriting recognition. The offline recognition [2,5,8,9,10], however, is still important and necessary, particularly in scenarios where the strokes are not available, such as whiteboards, historical documents, handwritten manuals, and printed files. In this paper, we focus on offline recognition and understanding. We aim at domains featuring the following structure: flowcharts consist of symbols connected by arrows, and there might be text labelling the symbols (the text is inside or along the border of a symbol) or arrows (the text is in a vicinity of an arrow). Although this structure is basic, there are various domains which fit it perfectly [2,3,4].

For recognizing symbols and arrows within a diagram and understanding its structure, object detection models developed in convolutional neural networks (CNN) can be employed to facilitate this task. While existing models can identify the individual objects like arrows and symbols, they lack the mechanics that are capable of obtaining a diagram’s structure understood. Such a shortage may be inconvenient to digitalization or reconstruction of a diagram from detection results on its hand-drawn version [3,7,9,10,11]. In this paper, we propose DrawnNet, a keypoint-based detector, which is based on recent state-of-the-art techniques in CNN. DrawnNet is designed upon CornerNet [12], where we explore extending CornerNet by introducing new modules that can effectively use explicitly prior knowledge existing in diagrams, making the promoted network accustomed to hand-drawn diagrams’ recognition tasks. Specifically, we propose two novel keypoint pooling modules which serve to explicitly embed prior knowledge like geometric characteristics existing in diagrams and then aggregate them into keypoint prediction. In addition, for understanding the diagram structure an arrow orientation prediction branch is proposed, which aims to predict which direction an arrow points to through predicting arrows’ head and rear keypoints.

**Contributions:**

- We propose a unified CNN-based keypoint-based detector DrawnNet to enable offline hand-drawn diagram recognition, which can not only accurately recognize individual symbols but also understand the whole structure of diagrams through arrow connections.
- Two novel keypoint pooling module are proposed, which are expected to explicitly encode and aggregate geometric characteristics within diagrams for various keypoint prediction.
- An arrow orientation prediction branch is proposed to enable diagram structure understanding through predicting which direction each arrow points to.
- Experiment results show that DrawnNet achieves 2.4%, 2.3%, and 1.7% recognition rate improvements compared with the state-of-the-art methods across benchmarks of FC-A, FC-B, and FA, respectively, outperforming existing diagram recognition systems on each metric.

The paper is organized as follows: Section 2 briefly surveys related work in diagram recognition and object detection. Section 3 describes our DrawnNet and its extensions. Section 4 presents experimental setting. Section 5 contains experimental results and analysis. Section 6 presents conclusions and future work.

## 2. Related Work

**Figure 1:**
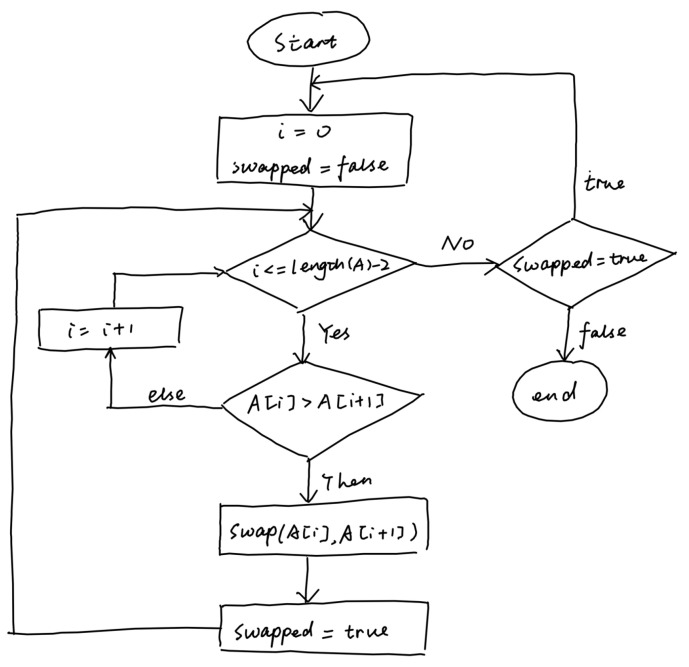
An example of a hand-drawn diagram.

This work focuses on handwritten diagrams, where a diagram consists of symbols, arrows, and optionally text phrases as shown in Figure 1. Usually, a symbol represents the semantics of diagram, each arrow connects two symbols representing the relationship between them, and each text annotates either a symbol or an arrow. Although this structure is simple, it is sufficiently powerful to describe graphical modeling languages from various domains. We follow the terminology in [2] and use the terminology arrow-connected diagram to refer to this kind of diagram.

### 2.1. Diagram Recognition

Handwritten diagram recognition methods can be grouped into two categories: online-targeted and offline-targeted recognition. For online recognition, the diagrams are drawn with an ink input device such as a tablet. This input device captures the drawing as a temporal sequence of strokes [2,5,8,9,10]. Online diagram recognition has received a lot of attention in research, especially in the area of flowcharts. However, these approaches are of limited applicability if the original stroke data are not available (e.g., hand-drawn diagrams on paper). While offline recognition directly allows for tackling this more general scenario, it has attracted much less attention in the past. Most offline approaches rely on traditional image processing methods to reconstruct the strokes of a diagram, and use feature engineering to derive a set of distinctive stroke features.

#### 2.1.1. Online Recognition

In the area of handwritten diagram recognition, many research works were conducted after the release of the Online Handwritten Flowchart Dataset (OHFCD) in 2011 by Awal et al. [13]. Lemaitre et al. [8] used a grammatical method to analyze the structure of the flowcharts. Carton et al. [14] further incorporated statistical information into this method. Bresler et al. [9] proposed a pipeline where they first extracted symbol candidates and then used a max-sum model to solve the optimization task of finding the best set of symbol candidates. Symbol candidates are generated by grouping temporally and spatially close strokes. In later work [2,5,10], their pipeline got improved by such as introducing a text classifier.

#### 2.1.2. Offline Recognition

Existing offline diagram recognition methods can be further divided into two groups: stroke-based [2,7] and object-based [6,11]. Stroke-based methods assume that the strokes in an image can be reconstructed in a preprocessing step. For example, some research either binarizes the image using a constant threshold [2], or recognizes a simplified diagram by the ground-truth strokes during inference [7]. After stroke reconstruction, previous works consider strokes in spatial approximation. Wu et al. [7] proposed shape estimation to induce if a stroke grouping has a regular appearance. Bresler et al. [2] also put up with an offline extension that uses a stroke reconstruction preprocessing step. Wang et al. [3,4] trained a max-margin Markov random field on stroke features to carry out segmentation and recognition. In addition, Bernhard et al. [6] proposed an object-based method by directly detecting diagram symbols using deep learning object detectors. However, what detectors they employed are not naturally targeted to diagrams. In other words, these detectors are designed toward general scenarios, and do not make full use of diagrams’ characteristics like shape.

### 2.2. CNN-Based Object Detection

In the area of object detection, recent work is mostly based on CNNs. Within this family, some detectors are anchor-based, which set anchor boxes in each position of the feature map. The network predicts the probability of having objects in each anchor box and adjusts the size of the anchor boxes to match the object. R-CNN series [15,16] are typically anchor-based models, which first extract Region of Interest (RoI) using a selective search method and then classify and regress them. Faster R-CNN [16] employs a region proposal network (RPN) to generate RoIs by modifying preset anchor boxes. Mask R-CNN [17] replaces the RoIPool layer with the RoI-Align layer using bilinear interpolation. Its mask head uses a top-down method to obtain instance segmentation. Some methods directly classify and regress the preset anchor boxes without RoIs. SSD [18], YOLOs [19,20,21] utilize features maps from multiple different convolution layers to classify and regress anchor boxes with different strides.

Usually, the sizes of anchor boxes are required to be carefully designed to fit a variety of objects. However, anchor-free detectors no longer need anchor boxes. Some anchor-free detectors belong to so-called keypoint-based ones, which directly predict keypoints and group them to generate bounding boxes. For example, CornerNet [12] predicts top-left and bottom-right corners of the object and pairs corners of the same object by similarity between each pair of points. CenterNet [22] adds a center detection branch into CornerNet and largely improves the performance by center point validation. ExtremeNet [23] detects the top-, left-, bottom-, rightmost, and center keypoints of the object to form the bounding box. In addition, these extreme points can be further used for object segmentation [24]. RepPoints [25] uses Deformable Convolutional Networks(DCN) [26] to predict points for representing objects. These detectors all need some specific grouping methods to obtain bounding boxes.

Recently, researchers began to explore transformer-based [27] visual architectures in visual tasks for their powerful modeling interdependences. Visual transformers have performed the state-of-the-art across many visual tasks. ViT [28] is the pioneer of visual transformers [29], which is directly applied to image classification. In object detection, DETR [30] and Deformable DETR [31] all successfully utilize transformers through a CNN for visual encoding and transformers for decoding into detection outputs. In segmentation, TransUNet [32] concatenates a CNN with a transformer as an excellent encoder for medical image segmentation. SETR [33] is devised as a pure-transformer segmentation model through treating an input image as a sequence of image patches.

Visual transformers are typical encoder–decoder structures such as an hourglass [34], adopted as the backbone in this paper and U-Net [35] is widely used in image segmentation [36,37,38], image reconstruction [39], and now widely leveraged in visual tasks. However, transformer-based visual architectures have some inherent limits worth serious consideration when applying them. First of all, they usually require high-resolution image inputs when applied in the complex tasks such as detection or segmentation, which undoubtedly raises an immense amount of computation and memory exhaustion [28,31,40]. Thus, they may get involved in an overfitting embarrassment when they are applied in middle-scale or small-scale visual scenes like diagram recognition. In addition, then, visual transformers demand a large-scale training dataset (for example, JFT-300M in ViT) to converge [28,30,31], which is difficult to meet in diagram recognition where each diagram benchmark only supports less than 400 training images. Therefore, comprehensively considering limits above and problem-solve moderation, we follow CornerNet to continue hourglass [34] as the backbone in our DrawnNet to keep compatibility.

### 2.3. Feature Aggregation

Feature aggregation is usually done to leverage to refine feature information from different channel or spatial feature maps. There are many visual-task models which have been furnished with feature aggregation modules. For example, a feature pyramid network [41] was proposed to aggregate multi-scale objects’ features by concatenating a pyramid of down-sampled convolution features.

Nowadays, many feature aggregation modules are built on so-called visual attention, which was derived from the feature integration theory [42]. Channel attention is a wide-known feature aggregation module, which was first proposed in SENet [43] to explicitly exploit inter-channel relationships. To balance performance and complexity, ECA-Net [44] is proposed as an efficient channel attention module, which only involves a handful of parameters while bringing clear performance gain. Ref. [45] devises a pyramid attention structure for salient object detection through aggregating multi-scale saliency information. In particular, content-based image retrieval can benefit from visual attention mechanisms by aggregating features from images (target and query) [46] or multiple-level [47,48] into attention maps such as region of interest or saliency information.

In image segmentation, DANet [49] combines self-attention and channel attention to capture rich contextual dependencies, and A2-FPN [50] proposed attention modules to enhance the feature pyramid network for the improvement of multi-scale feature learning through for the attention-guided feature attraction and aggregation. In image super-resolution, channel attention is introduced into deep CNNs to further improve super-resolution performance [51,52].

In addition, there are some aggregation algorithms based on pooling methods. CBAM [53] is composed of both spatial and channel-wise attention modules, which leverages both global average and max pooling to aggregate features. GSoP [54] introduces a second-order pooling to enhance capability of nonlinear representation learning for more effective feature aggregation. GE [55] explores spatial extension using a depth-wise convolution to aggregate features.

Our feature aggregation measures adopted in DrawnNet are based on pooling methods, where the proposed keypoint pooling methods are first applied in each branch’s feature maps along the channel to make full use of the geometric information in the image, then the pooled maps from different pooling method are aggregated through multiplying, summarizing or concatenating each other for adaptive feature refinement.

## 3. DrawnNet for Diagram Recognition

### 3.1. Network Architecture

In DrawnNet, we detect each symbol in diagrams as a pair of keypoints, namely the top-left corner and bottom-right corner that collectively determine a bounding box. In addition, for each arrow that serves to connect any of two symbols, there is a branch called arrow orientation prediction designed for predicting the head and rear keypoints of arrows, which tells us to which direction an arrow points. Through this branch, a diagram’s structure can be understood completely.

**Figure 2:**
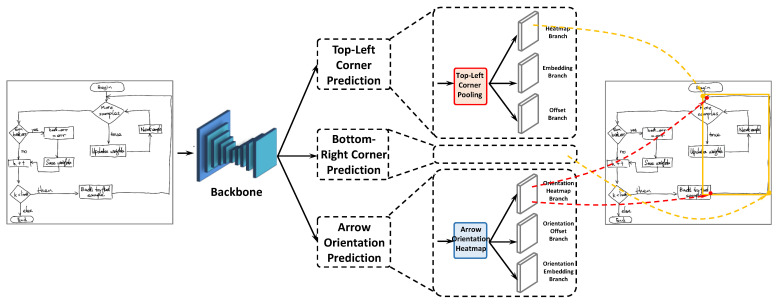
The architecture of DrawnNet. A backbone with the structure of encode–decode is followed by two keypoint prediction branches (heatmap, embedding, and offset) for top-left and bottom-right corner prediction, respectively, and one arrow orientation prediction branch for keypoints of arrow heads and rear prediction.

Figure 2 provides an overview of DrawnNet. We employ the hourglass network [34] as the backbone network of DrawnNet as the same as CornerNet [12]. Hourglass is a typical encoder–decoder structure, which has been widely applied in keypoint detection tasks like pose estimation [34,56]. In DrawnNet, the backbone is succeeded by three parallel prediction branches, two of which are responsible for top-left and the bottom-right corner keypoint prediction, and the third takes on the mission of predicting the head and rear keypoints of arrows. Each branch is furnished with its own keypoint pooling modules and feature aggregation modules to explicitly embed geometric characteristics within diagrams into feature maps derived from the backbone. This could definitely help refine and augment keypoint semantic information in feature maps before they are passed to later convolutional layers for heatmaps, embeddings, and offsets.

Therefore, we propose two keypoint pooling modules to enrich keypoint information, and an arrow orientation prediction branch to enable arrow orientation prediction. The first pooling module, Cumulative Intersection Corner Pooling(CICP) used in top-left and bottom-right corner keypoint prediction branches, is designed from CornerNet [12]’s pooling method referred to as Maximal Intersection Corner Pooling (MICP) in this paper. CICP aims to exploit more recognizable visual corner patterns lying in intersections of symbol boundary lines which vertically or horizontally move forward, making the model easier to perceive corner keypoints in rectangular shapes. The second pooling module is Snow Corner Pooling (SCP), which is installed in the arrow orientation prediction branch and is targeted at capturing arrow head and rear keypoints. Because arrow keypoints are usually cross-like patterns present in diagrams and point somewhere at one of four orientations (upward, downward, leftward and rightward), SCP would slide over feature maps, probing toward eight directions to match arrows, which expands like snow and hence is named.

### 3.2. Corner Keypoint Prediction

**Figure 3:**
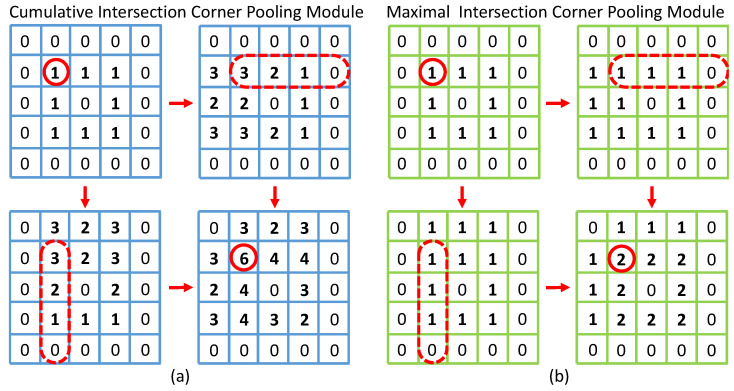
CICP and MICP are leveraged to pool the top-left corner appearing in the same rectangle; (**b**) obviously demonstrates that MICP fails to capture the top-left corner. By contrast, (**a**) with CICP is adequate to tackle such situation.

In DrawnNet, an object is represented as a pair of keypoints, namely top-left and bottom-right corners. However, as shown in Figure 3, there is often a lack of local visual evidence, which could indicate where corners present. In CornerNet [12], to locate the latent corners, the authors proposed a pooling module referred to as Maximal Intersection Corner Pooling (MICP) in this paper. The module maxpools horizontally and vertically from a pixel to look for the maximums along these two directions and then add them up. In diagrams, most of the symbols are rectangular contours and their corners apparently present where several boundaries intersect with each other. Thus, in DrawnNet, we extend the original corner pooling by the introduction of another pooling-reduce method to artificially encode into corner keypoint prediction these geometric characteristics exposed in diagrams as explicit prior knowledge.

In CornerNet [12], corner pooling computing goes through each pixel horizontally and vertically. If one of the neurons responds most strongly within a neighborhood in a pooled feature map, the location could be a latent corner, and it is placed at the intersection of the horizontal pooling vector and vertical pooling vector. We refer to this kind of corner pooling as Intersection Corner Pooling (ICP). In CornerNet, the authors adopted max as ICP’s reduction called Maximal Intersection Corner Pooling (MICP) to compute the final response, whereas, in DrawnNet, we use sum to accumulate all responses along vertically and horizontally as ICP’s reduction called Cumulative Intersection Corner Pooling (CICP).

#### 3.2.1. Intersection Corner Pooling

As shown in Figure 3, MICP and CICP are leveraged respectively to pool the same feature map, in which they are expected to capture the top-left corner belonging to a rectangular-like pattern composed of responses having a numerical value of 1. Such rectangular-like patterns are undoubtedly fundamental contours in diagrams. Figure 3b obviously demonstrates that MICP fails to capture the top-left corner(circled by red solid line) for other neurons within its neighborhood in the pooled map respond with almost the same magnitude as itself. By contrast, CICP in Figure 3a is adequate to tackle such a situation in the pooled map by letting the according neutron respond with the largest magnitude within its neighborhoods such as 3×3 and 5×5.

As described above, to determine if a pixel is a top-left corner, ICP would look horizontally towards the right for the topmost boundary of an object and vertically towards the bottom for the leftmost boundary.

Let $F_{t}$ and $F_{l}$ be the feature maps that are the inputs to corner pooling layer, and let $F_{t_{ij}}$ and $F_{l_{ij}}$ be the responses at location $\left(i,j\right)$ in $F_{t}$ and $F_{l}$, respectively. With H × W feature maps, CICP determines if a pixel at location $\left(i,j\right)$ is a top-left corner through in parallel accumulating all responses horizontally distributed in $\left(i,j\right)$ and $\left(i,H\right)$ in $F_{t}$ and vertically distributed in $\left(i,j\right)$ and $\left(W,j\right)$ in $F_{l}$ into the sum $T_{ij}$ and $L_{ij}$, respectively. Finally, it adds them into $F^{CICP}$. The computing process can be articulated by the following formulas:

(1) $$T_{ij}=\sum_{k=i}^{H}max\left\{0,F_{t_{kj}}\right\}$$

(2) $$L_{ij}=\sum_{k=j}^{W}max\left\{0,F_{l_{ik}}\right\}$$

CICP for the bottom-right corner is computed in a similar way to top-left corner. It accumulates in parallel all responses vertically distributed in $\left(0,j\right)$ and $\left(i,j\right)$ and horizontally distributed in $\left(i,0\right)$ and $\left(i,j\right)$ before adding the pooled results. The corner pooling layers are used in the prediction modules to predict heatmaps, embeddings, and offsets.

#### 3.2.2. Geometric Characteristics Aggregation

**Figure 4:**
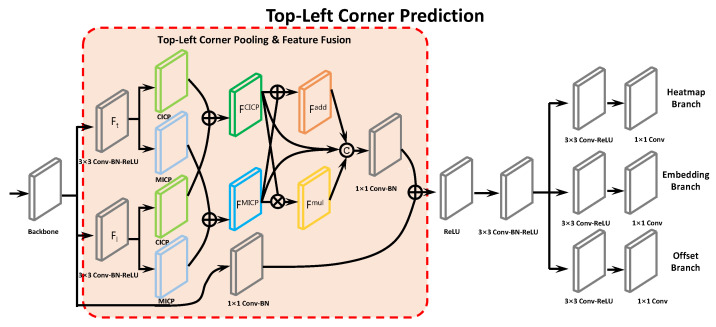
The architecture of the top-left corner prediction branch with CICP and MICP, and geometric characteristics aggregation.

The architecture of the top-left corner prediction branch is shown in Figure 4. Our improvements compared to CornerNet comprise the extension to its corner pooling module and the additional introduction to an aggregation of what features multiple pooling modules capture. We design CICP(as described above) as a complement to MICP to enrich the corner feature particularly. Referring to the residual block [57], we construct the whole corner pooling & feature fusion module by replacing the first 3×3 convolution module with two 3×3 Conv-BN-ReLU layers with 128 channels in parallel to process the features from the backbone. Then, MICP and CICP are applied in parallel to pool these two feature maps (for example, $F_{t}$ and $F_{l}$ for the top-left corner), where one is prepared for vertical pooling and the other for horizontal before their respective pooled maps are added up as results $F^{CICP}$ and $F^{MICP}$.

It is important to note that some corners may be captured by one of CICP and MICP but not effectively by the other, and vice versa as demonstrated in Figure 3. Therefore, it is necessary to aggregate what these two pooling modules capture to make them complement each other. Sequentially, we aggregate $F^{CICP}$ and $F^{MICP}$ by element-wise addition and element-wise production into $F^{add}$ and $F^{mul}$, respectively. Then, like what is adopted in Inception [58] for aggregating features from multiple Conv-Pooling layers, we concatenate $F^{CICP}$, $F^{MICP}$, $F^{add}$, and $F^{mul}$ together into a chunk of feature maps, which is later fed into a 1×1 Conv-BN layer with 256 channels for channel reduction. Finally, we add back the the output with the shortcut passed from previous backbone through a 1×1 Conv-BN layer with 256 channels too before going through a ReLU layer for nonlinear transformation.

The modified residual block is then followed by a 3×3 Conv-BN-ReLU layer with 256 channels before generating three parallel branches to produce the heatmaps, embeddings, and offsets, each of which goes through a 3×3 Conv-BN-ReLU layer again and a 1×1 Conv layer for different channels.

### 3.3. Arrow Orientation Prediction

Structure recognition in arrow-connected diagrams like flowcharts involve specifying which symbols each arrow connects and which directions each arrow points to. While an object detector can classify and localize the symbols of a diagram through bounding boxes, this information is insufficient for structure recognition. We found that this problem can be effectively tackled through arrow keypoint information.

**Figure 5:**
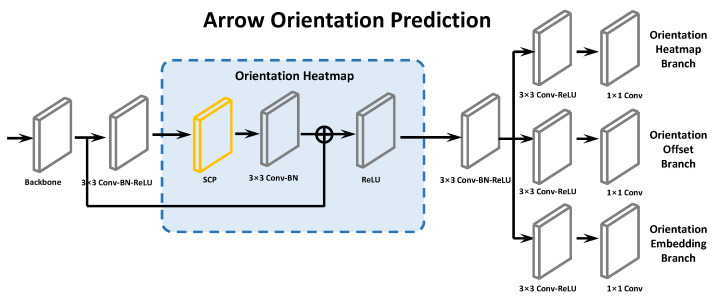
Arrow orientation Prediction with SCP to facilitate structure recognition.

For predicting arrow keypoints, we add a parallel arrow orientation prediction branch to the backbone. Figure 5 shows the network. The arrow network reuses the feature maps from the backbone and use SCP to enhance the arrow keypoint information.

#### 3.3.1. Snow Corner Pooling

**Figure 6:**
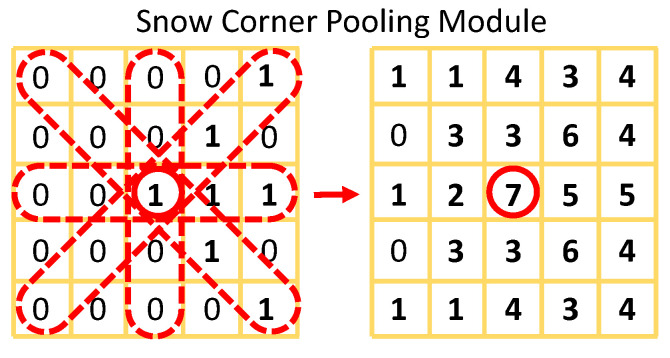
An SCP example with r=2, which demonstrates how SCP is leveraged in the arrow orientation branch to capture an arrow pattern.

Arrow orientation can be determined through locating arrows’ heads and rears, which can be detected as a keypoint detection task through the inherent aptitude of DrawnNet. To address this issue, we propose Snow Corner Pooling (SCP) to capture richer and more recognizable visual arrow patterns. Figure 6 shows the principle of SCP.

Let *F* be the feature maps for SCP, and let $F_{i,j}$ be the responses at location $\left(i,j\right)$ in *F*. With H×W feature maps, the response at location $\left(i,j\right)$ in the pooled feature map $F^{SCP}$ through SCP can be articulated by the following formulas:

(3) $$F_{i,j}^{SCP}=max\left\{0,F_{i,j}\right\}+\sum_{n=1}^{r}\left[\sum_{p\in \left\{-n,n,0\right\}}^{}\sum_{q\in \left\{-n,n,0\right\}}^{}max\left\{0,F_{i+p,j+q}\right\},\left|p\right|+\left|q\right|\neq 0\right]$$

where *r* is the pooling radius, which configures how large the pooling scope is. Figure 6 shows that SCP is exerted on an arrow pattern, which successfully captures the arrow keypoint by letting the neutron respond most intensely.

#### 3.3.2. Arrow Orientation Prediction

In DrawnNet, similar to the corner prediction branch, in an arrow orientation prediction branch, we predict arrow head and rear keypoints using heatmaps, offsets, and embeddings. Here, heatmaps with size H×W have *C* channels, where *C* is the number of categories and is set to 2, indicating whether an arrow keypoint is a head or rear. Let $p_{cij}$ be the probability at location $\left(i,j\right)$ for class *c* in the predicted heatmaps, and let $y_{cij}$ be the “ground-truth” heatmap. Then, the category loss for arrow keypoints can be estimated through focal loss [59]:

(4) $$L_{det}^{arr}=\frac{-1}{N}\sum_{c=1}^{C}\sum_{i=1}^{H}\sum_{j=1}^{W}\left\{\begin{array}{cc} {\left(1-p_{cij}\right)}^{\alpha }\log\left(p_{cij}\right) & \;\mathrm{if}\;y_{cij}=1\\ {\left(p_{cij}\right)}^{\alpha }\log\left(1-p_{cij}\right) & \mathrm{otherwise} \end{array}\right.$$

where *N* is the number of objects in an image, and α is the hyper-parameters which control the contribution of each point (we set α to 2).

It is common that convolutional networks would downsample inputs to refine global semantic information, which definitely results in smaller resolutions of outputs than those of inputs while reducing memory usage. This means that some position precision may become impaired when these locations from the heatmaps are remapped onto the input image. In DrawnNet, offsets are predicted to slightly rectify the arrow keypoint locations. Let $\left(x,y\right)$ be a location in the image and $\left(\left\lfloor \frac{x}{s}\right\rfloor ,\left\lfloor \frac{y}{s}\right\rfloor \right)$ is its downsampled location in the heatmaps, where *s* is the downsampling factor. The deviations for the arrow keypoint *k* between these two locations can be estimated:

(5) $$o_{k}=\left(\frac{x_{k}}{s}-\left\lfloor \frac{x_{k}}{s}\right\rfloor ,\frac{y_{k}}{s}-\left\lfloor \frac{y_{k}}{s}\right\rfloor \right)$$

Here, Smooth L1 Loss is used to evaluate the errors between predicted values and ground-truth:

(6) $$L_{off}^{arr}=\frac{1}{N}\sum_{k=1}^{N}Smooth-L1Loss\left(o_{k},{\hat{o}}_{k}\right)$$

A diagram may include more than one arrows, and thus multiple head and rear keypoints may be predicted. Therefore, it is necessary to determine which pair of head and rear keypoints belongs to the same arrow. Our approach is also associative embedding used in CornerNet to group up two keypoints which have the largest similarity. Let $e_{hk}$ be the embedding for the head keypoint of arrow *k* and $e_{tk}$ for the rear keypoint, where they are all four-dimensional vectors. As in corner prediction, we also use the “pull” loss to train the network to group the keypoints and the “push” loss to alienate the keypoints:

(7) $$Sim\left(e_{1},e_{2}\right)=\frac{\langle e_{1},e_{2}\rangle }{\left|e_{1}\right|\cdot \left|e_{2}\right|}$$

(8) $$L_{pull}^{arr}=\frac{1}{N}\sum_{k=1}^{N}\left[1-Sim\left(e_{tk},e_{hk}\right)\right]$$

(9) Lpusharr=1N(N−1)∑k=1N∑j=1j≠kNSimetk,ehj

where Sim is a similarity metric, and we only apply the losses at the ground-truth corner location.

Finally, all branch loss functions are linearly combined to form the total loss function, in which branch loss functions with the same property share the same coefficient as follows:

(10) $$L=L_{\det\;}^{\mathrm{arr}}+L_{\det}+\alpha \left(L_{\mathrm{pull}\;}^{\mathrm{arr}}+L_{\mathrm{pull}\;}\right)+\beta \left(L_{\mathrm{push}\;}^{\mathrm{arr}}+L_{\mathrm{push}\;}\right)+\gamma \left(L_{\mathrm{off}}^{\mathrm{arr}}+L_{\mathrm{off}}\right)$$

where $L_{\det}^{\mathrm{arr}}$, $L_{\mathrm{pull}\;}^{\mathrm{arr}}$, $L_{\mathrm{push}\;}^{\mathrm{arr}}$ and $L_{\mathrm{off}}^{\mathrm{arr}}$ are losses for arrows and $L_{\det\;}$, $L_{\mathrm{pull}\;}$, $L_{\mathrm{push}\;}$ and $L_{\mathrm{off}}$ are losses for corners as described in CornerNet [12]. α, β, and γ denote the weights for corresponding sub-task losses and are set to 0.1, 0.1, and 1, respectively. Meanwhile, we find one or larger values of α and β lead to the poor convergence.

## 4. Experiments

### 4.1. Training

Our method is implemented in Pytorch [60] and the network is trained from scratch. The resolution of the input image is 511×511, leading to heatmaps of the size 128×128. We use the data augmentation strategy presented in [6,12] to train a robust model. Adam [61] is used to optimize the training loss. We train DrawnNet on 4 Tesla V100 (16GB) GPUs and use a batch size of 16. The maximum number of iterations is 20 K. We use a learning rate of $2.5\times 10^{-4}$ for the first 30K iterations and then continue training 5K iterations with a rate of $1.5\times 10^{-5}$.

### 4.2. Inference

During inference, we follow CornerNet [12] to select the top 100 top-left and top 100 bottom-right corners from the corner heatmaps and 100 heads and 100 rears from arrow orientation heatmaps. The corner and arrow locations are adjusted by the corresponding offsets. For top-left and bottom-right corners, we measure the L1 distances between their embeddings and for heads and rears, we measure Sim between their embeddings. Pairs that have unqualified measurements (greater than 0.5 for corners and less than 0.5 for arrows) or contain partners from different categories are abandoned. The average score of each pair is considered as its detection score.

The common NMS [62,63] has the inherent prejudice to recognizing symbols in diagrams: it generally believes that there is little overlap between two bounding boxes with the same category, and when the IoU between them is greater than a threshold (for example, 50%), it would think that they are predicting the same object. Thus, this will inevitably result in only one of them remaining and the other will be considered redundant and get filtered out. However, in diagrams, there are many situations in which one bounding box has a degree of overlap with the other. For instance, flowcharts and finite automata allow arrows connecting two symbols, where bounding boxes of arrows are endowed with a large magnitude of overlap, especially for opposite arrows that connect the same symbol. Therefore, we employ NMS proposed in [6] instead of that used in CornerNet [12].

For each predicted arrow, it is required to specify which symbols they link. Following [6,7], we appoint those symbols that are closest to each arrow’s keypoints (heads or rears), where the closeness is defined as the distance between a keypoint and a symbol bounding box.

### 4.3. Datasets

We evaluate DrawnNet on three public handwritten diagram datasets, two of which depict flowcharts (FC_A and FC_B) and one is a finite automata dataset (FA).

FC_A [13] was released in 2011 as a benchmark database of flowcharts, which consists of 419 diagrams (248 for train split and 171 for test split) drawn by 35 writers from 28 predefined templates.The biggest deficiency among this database is the lack of annotations about the diagram structure and temporal information. Only individual symbols are provided. Thus, the data are of low quality and hardly used to evaluate online methods.

FC_B [5] was published as a complement for FC_A in 2016, which contains 672 samples (280 for train split, 196 for test split, and 196 for validation split) derived from 28 pattern templates drawn by 24 writers. Some of the templates refer to FC_A, and the rest include common-used algorithm functionality. In addition to diagram structure annotations, its annotations include arrow pointing directions.

FA was to be made public at the same time with FC_B in [5], which has a total of 300 diagrams (132 for train split, 84 for test split and 84 for validation split) generated from 12 pattern templates sketched by 25 writers. The dataset has four categories: state (a single circle), final state (two concentric circles), test, and arrow. Arrows are typically curved, except the initial arrow. Just like FC_B, its annotations also include arrow pointing directions.

It is necessary to mention that the three datasets above are originally used for online recognition, which means that each diagram is recorded as a sequence of strokes coordinates instead of a digital image. Thus, it is required to convert them into corresponding offline editions. For the FC_B dataset, we use the offline FC_Bscan dataset introduced in [5], which contains scans of printed FC_B diagrams. For the other two datasets, thanks to Bernhard et al. [6], they rendered diagrams into images and annotated bounding boxes as well as arrows’ keypoints at https://github.com/bernhardschaefer/handwritten-diagram-datasets, accessed on 16 November 2021.

### 4.4. Evaluation Metrics

We evaluate our method using recognition metrics on the symbol and diagram level. Regarding symbol recognition, Bresler et al. [2,5,9] compute the symbol recognition recall at an IoU threshold of 80%. Additionally, arrows are required to be connected to the correct symbols. When using an object detector, the recall negatively correlates with the specified detection score threshold. To make the symbol recognition recall comparison somewhat fair, Bernhard et al.[6] use a score threshold of 0.7 for postprocessing throughout all their experiments. We employ the same configuration with them in our experiments.

On a more aggregate level, the diagram recognition metric intuitively assesses the performance of a diagram recognition system as the ratio of correctly recognized diagrams in a test dataset, where a diagram has been recognized if the number of detected symbols equals the number of ground-truth symbols, each symbol has been correctly classified and localized with at least the IoU 80%, and each of the arrows has connected correctly.

## 5. Evaluation Analysis

### 5.1. Diagram Recognition

**Figure 7:**
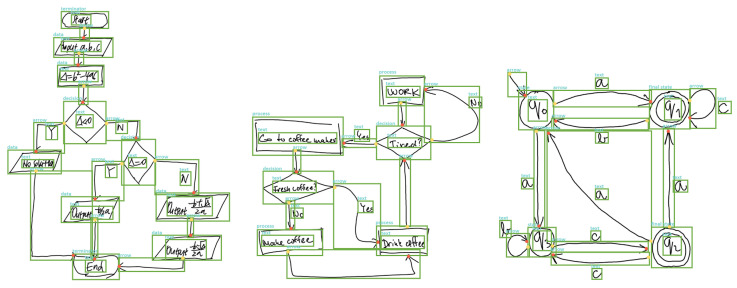
Some diagrams’ recognition by DrawnNet from the test split of three benchmarks. Here, arrow heads and rears are marked with red and yellow dots, respectively.

Table 1 shows that DrawnNet is evaluated compared with other online and offline recognition systems. For online recognition systems, they run based on analysing and modeling temporal stroke information [2,5,7,9,11,64], which to a large extent leverage symbol segmentation and symbol classification. These low-level representation and local understanding are sensitive to benchmarks and may cause a negative impact on recognition precision if annotations are low-quality or imprecise [2,5,9]. Instead, recognition systems powered by deep learning can robustly reach an excellent performance in diagram recognition task through target-designed networks. Figure 7 shows DrawnNet performs on the test split of three benchmarks.

### 5.2. Symbol Recognition

We also reveal how DrawnNet performs on symbol-level recognition across each benchmark. Table 2, Table 3 and Table 4 show symbol recognition results for each benchmark. Overall, DrawnNet achieves perfect recognition results for several node shapes, which can be explained by the fact that the shape and scale of nodes has a much lower variance than arrow and texts.

**Figure 8:**
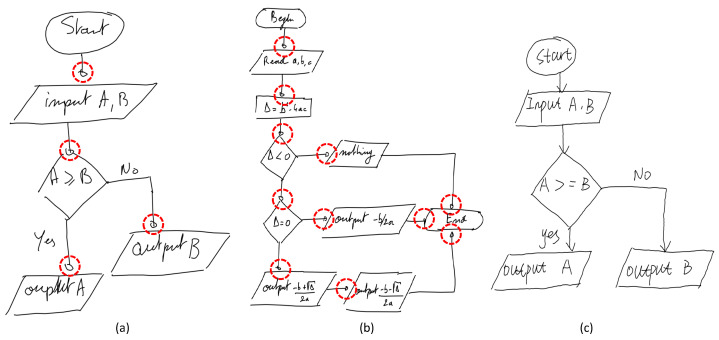
Some bug samples in a training split of $FC_{A}$. (**a**,**b**) show arrow heads are mismarked with circles and too small; (**c**) shows a normal sample.

On the FC_A dataset (Table 2), DrawnNet has a much higher symbol recognition recall and precision. However, DrawnNet performs slightly inferiorly on Arrow category across all categories. Through our examination on the train split of FC_A, we found that there are some samples where arrow heads are marked with circles instead of cross-shapes, which may hinder the model’s classification decision and make it confused with other categories like text, misleading the model learning as shown in Figure 8a. In addition, another problem exists in that some of these circles are too small in input resolution, which would become smaller and smaller after a series of downsampling in feature maps as shown in Figure 8b. Thus, it seriously hinders feature extraction conducted by our corner pooling modules as articulated earlier.

Table 3 shows that DrawnNet can accurately recognize symbols in scanned diagrams. It is interesting that Arrow R-CNN [6] gives a complementary result on precision and recall of Data and Process categories, which means its precision and recall on the category Data are 100 and 94.9, respectively, but, for the Process, the result is almost the reverse. This is partly because, despite the employment of FPN [41], Arrow R-CNN’s underlying network Faster R-CNN [16] is not equipped with the mechanisms to learn fine-grained distinctive information between similar objects with different categories [65,66], where symbols of Data and Process are all quadrilateral shapes except that the two parallel edges of Data symbols are slightly tilted instead of being vertical like Process symbols.

As Table 4 illustrates, DrawnNet perfectly recognizes the state and final state shapes in the FA test split. Because the categories of this benchmark are relatively small and the features of each category are very stable, it is not difficult to recognize.

### 5.3. Ablation Study

Finally, we conduct ablation study across each benchmark to further quantify the effect of our proposed keypoint pooling methods on diagram recognition. Table 5 shows the ablation results where the combination of CICP and SCP can substantially improve the rate of diagram recognition. Here, we should point out that the arrow orientation branch is proposed in this paper, but it can not be dismissed in the ablation study for its responsibility of predicting arrow keypoints, which is indispensable for diagram recognition. Thus, we just ablate SCP used in arrow orientation branch in the ablation study instead of the whole branch network.

It can be obviously seen that the employment of SCP effectively improves the rate of diagram understanding, for it is targeted to help predict arrow keypoints for its fantastic pooling fashion. Whether arrow keypoints are predicted is fundamental to later diagram recognition. Additionally, CICP also help DrawnNet perceive where corner keypoints in rectangular contours may be located, but it may not perform its potential so well if used alone. After all, whether arrow keypoints are predicted correctly is critical to the correctness of diagram understanding.

## 6. Conclusions

In this paper, we propose DrawnNet, a keypoint-based detector for offline handwritten diagram recognition. DrawnNet performs state-of-the-art on both diagram recognition and symbol recognition across each benchmark. We show that the keypoint-based detector can be appropriate to recognize hand-drawn diagrams by the way of designing targeted pooling modules to explicitly embed into feature learning the prior knowledge like geometric characteristics existing in diagrams’ symbols. Since standard CornerNet lacks the capability of predicting arrow orientation, we furnish DrawnNet with an arrow orientation branch, parallel to corner keypoint prediction branches, which is responsible to predict where arrow head and rear keypoints are.

Our study provides a new perspective that hand-drawn diagrams such as flowcharts and finite automate, which consist of polygonal contours such as rectangle, square, diamond, and circle, can be recognized and understood effectively through predicting some of the keypoints such as top-left corner, bottom-right corner, arrow head, and arrow rear. In future work, we would like to adapt DrawnNet to support other diagrammatic domains beyond the scope of arrow-connected diagrams. For example, we plan to extend the proposed method to recognize algorithm flowcharts, which definitely contain more complicated structures like nested control flows, which are expected to express high-level program semantics such as loop, iteration, jump, and select. Once algorithm flowcharts are recognized, we could generate corresponding code through parsing recognized results, which will extremely reduce the work of manual coding and improve productivity.

## Figures and Tables

**Table 1:** Diagram recognition rate across each benchmark.

	FC_A	FC_B	FA
Wang et al. [4]	5.8	-	-
Julca-Aguilar et al. [64]	34.0	-	-
Martin Bresler et al. [2]	-	37.7	-
Martin Bresler et al. [5]	59.1	67.9	79.8
Bernhard Schafer et al. [6]	68.4	78.6	83.3
DrawnNet	70.8	80.9	85.0

**Table 2:** FC-A symbol recognition at IoU 0.80 on test split.

	[7]	[10]	[3]	[6]		DrawnNet	
**Class**	**Recall**	**Recall**	**Recall**	**Precision**	**Recall**	**Precision**	**Recall**
Arrow	80.3	74.4	83.4	94.7	96.0	95.7	97.1
Connection	73.4	93.6	79.8	99.2	100	99.6	100
Data	78.5	91.7	84.4	100	99.7	99.9	99.8
Decision	78.9	74.1	76.9	100	99.5	100	99.7
Process	88.3	87.2	89.2	99.8	100	100	100
Terminator	90.6	88.1	80.8	100	100	100	100
Text	86.0	87.9	85.8	99.3	99.1	99.3	99.1
Total	83.2	82.8	84.3	97.9	98.3	98.4	98.8

**Table 3:** FC-B symbol recognition at IoU 0.80 on test split.

	[2]		[6]		DrawnNet	
**Class**	**Precision**	**Recall**	**Precision**	**Recall**	**Precision**	**Recall**
Arrow	85.1	84.3	98.0	98.0	98.6	98.7
Connection	61.0	86.6	100	100	100	100
Data	79.7	94.4	100	94.9	100	95.8
Decision	83.2	96.9	100	100	100	100
Process	88.6	98.8	95.5	100	96.4	100
Terminator	71.9	93.6	100	100	100	100
Text	99.5	93.7	99.2	99.3	99.5	99.5
Total	95.0	91.3	98.7	98.7	99.0	99.1

**Table 4:** FA symbol recognition at IoU 0.80 on test split.

	[10]	[3]	[6]		DrawnNet	
**Class**	**Recall**	**Recall**	**Precision**	**Recall**	**Precision**	**Recall**
Arrow	84.4	95.3	98.4	98.4	98.6	98.7
Final state	93.8	89.1	100	100	100	100
State	94.5	91.2	100	100	100	100
Text	96.0	98.1	99.6	99.7	99.6	99.7
Total	92.2	95.8	99.3	99.3	99.5	99.4

**Table 5:** Ablation study across each benchmark.

CICP	SCP	FC-A	FC-B	FA
-	-	68.8	78.8	83.7
*√*	-	69.5	79.1	84.1
-	*√*	70.2	80.3	84.3
*√*	*√*	70.8	80.9	85.0

## Data Availability

Data sharing is not applicable for this article.

## Abbreviations

The following abbreviations are used in this manuscript:

CNN	Convolutional Neural Networks
DCN	Deformable Convolution Networks
RoI	Region of Interest
ICP	Intersection Corner Pooling
CICP	Cumulative Intersection Corner Pooling
MICP	Maximal Intersection Corner Pooling
SCP	Snow Corner Pooling
